# Interesting Analyses of Articles in Foreign Journals

**Published:** 1864-08

**Authors:** 


### INTERESTING ANALYSES OF ARTICLES IN FOREIGN JOURNALS.

*Life-value of Premature Labor.*—The distinguished Obstetrician, Edmund A. Kerby, M.D., etc., on remarking upon the value of inducing premature labor, as an advantage over craniotomy, produces the result of his experience in the following statistics: in craniotomy, one mother in five dies; while in the induction of premature labor there is a loss of only one mother in fifty.

*Death from Inhalation of Chloroform.*—The *Gazette Médicale*, of Lyons, reports another death from the inhalation of chloroform.

*Resection.*—M. Maisonneuve, of the Surgeons of La Pitié, laid before the Academy of Sciences, at its last meeting, the notes of a case in which the advocates of conservative surgery cannot fail to be much interested. A young man, in the month of August, 1855, consulted this practitioner in consequence of an extensive necrosis of the tibia of the right leg. The limb presented a deplorable aspect, its volume being triple that of the natural size, and the surface of the skin being furrowed by deep ulcers, through which the mortification of the whole bone during its entire length could be perceived. The disease had commenced after a fall, which occurred two years previously, and the excessive suppuration had occasioned constitutional disturbance of a nature so serious as to threaten life. The most eminent surgical authorities of Paris (M. Velpeau amongst them) had declared that amputation of the thigh could alone save life, and that any idea of preserving the limb was, to say the least, Utopian. In spite of such imposing authority, and confident in the regenerating powers of the periosteum, so positively demonstrated by M. Flourens, M. Maisonneuve determined on attempting the subperiosteal resection of the entire tibia, an operation to which the patient readily consented, and which was accordingly performed on the 24th of August, 1855. Chloroform having been administered, an incision fourteen inches in length was made down the front of the leg, the periosteum was divided, the bone laid bare, entirely *shelled out* from its fibrous envelope, and so removed *in toto*. With the exception of the two articular surfaces, the osseous shaft was found to be diseased throughout its whole length. The wound healed kindly, and by the fortieth day after the operation the patient was able to walk with the aid of crutches, as in a case of simple fracture; and so complete had been the subsequent regeneration of the tibia that, at the present time, the limb differs in no respect from its fellow, with which it has kept pace in growth, as to length, strength, and bulk; and the only trace which remains of the terrible operation, to which the patient is indebted for the possession of his leg, is the long cicatrix with which its anterior surface is indellibly scored. The extirpated tibia exhibited to the Academy by M. Maisonneuve measures in length twelve inches and a half; in breadth, at its upper part, an inch and a quarter; at its lower extremity one inch. The committee appointed to report on this remarkable case includes Messrs. Flourens, Milne Edwards, Velpeau, Cloquet, Jobert de Lamballe, Claude Bernard, and Longet; and their report is awaited with the greatest impatience.

*Pulverization of Water for Bathing Purposes.*—The St. Louis Hospital, of Paris, is especially organized for the treatment of diseases of the skin, and therefore possesses a very complete set of medicated baths, which have hitherto been extremely useful. The physicians of the hospital have, however, not been unmindful of the late improvements which have been proposed in balneology. It is well known that both M. Sales Girons and M. Mathieu (de la Drôme) have invented very ingenious machines, by which water is minutely divided and projected, forming a kind of mist, directed with some force upon the bathers. The machine of the latter inventor has, for some time past, been on trial at the St. Louis Hospital, under the superintendence of the six physicians attached to the institution; and it has been resolved to adopt M. Mathieu's *hydrofere*. This machine will surround the patient with very fine rain for one hour, and expend during the process, only four quarts of liquid. Thus may sea-bathing be now enjoyed very far inland, and mineral baths be administered at a great distance from the wells.

*The Relation of the Intellectual Functions to the Pons-Varolii.*—It is an interesting fact, that in examining the brains of two hundred and eighty-nine cases of lunatics, the pons-varolii was only observed to be affected in two instances. This testimony goes far to establish the fact that the intellectual functions are entirely independent of the pons-varolii.

*Complete Transposition of all the Thoracic and Abdominal Viscera.*—The following interesting facts were observed in a post-mortem examination of a lady who died at the advanced age of 85 years. On opening the thorax and abdomen, a complete transposition of all the organs presented itself. The heart lay with its base towards the left side of the spinal column, the apex pointing towards the right side, and reaching to the lower border of the fourth rib under the right mamma. The pulmonary cavity of the heart, which was also on the left side; the aorta and systemic ventricle to the right: so that not only was the heart reversed in position, but also in formation. The left phrenic vein was lying on the superior vena cava; the right innominate was seen passing over the aorta to the left, and emptying itself into the superior vena cava. The lungs were healthy, but old pleuritic adhesions existed on both right and left sides, especially the former. The larger lobe of the liver was in close proximity to the left ribs, the smaller lobes extending only slightly to the right of the sternum. The spleen was situated on the right side, just beneath the heart; œsophagus lying to the right of the aorta. The stomach was situated on the right side, with cardiæ extremity touching the ribs, and the pyloric end extending to the left side of the mesial line; sigmoid flexure of color was on the right side.

*Leprosy.*—The etiology of this disease has at last been established; chemical analysis, in connection with a long series of investigations, has proven it to be a true dyscrasia or disease of the blood. An altered condition of the blood, demonstrable by chemical analysis, accompanied the premonitory symptoms; the blood always containing an excess of albumen and fibrine. This fact is satisfactorily proved by examining the history of leprosy, as it has appeared in various countries. In Norway and Ireland, where the population inhabit small, close and dimly-lighted huts; where the people are unclean in their personal habits, breathe a moist, marine atmosphere and live on inferior food, this disease is yet frequent. During the middle ages, when the population had become too numerous to be sustained by the produce of the chase, and before the art of producing large supplies was understood, leprosy was common in all parts of Europe. It disappeared as soon as full supplies of fresh meats, vegetables and bread were produced. In Egypt, Palestine, Spain and Brazil, where the very poor dwell in dark and unventilated huts, and where want and dirt are conspicuously manifested, lepers are still to be found. This disease now occurs exclusively among the lower grades of society. The disease is not contracted by those enjoying good diet, and surrounded by good higiene conditions.

*The Effect of Forests on Atmospheric Temperature.*—M. Becquerel has just published the results of certain experiments instituted by himself for the purpose of ascertaining the effects produced by the presence of forests on the temperature of the surrounding country. He has found that trees heated during the day, by the sun's action, and cooling during the night under the influence of nocturnal radiation, give rise first to an ascending current of hot air, and secondly to a descending current of cold air, which lowers the temperature of the surrounding soil during the night and morning. This result, according to M. Becquerel, proves why in the vicinity of a forest the temperature is lower than in an open plain. The cutting of timber in any great quantities would consequently have the effect of rendering the summers warmer and winters less cold, by procuring the removal of a cause producing a like diminution of temperature in either season.

*Amputation at Hip-joint.*—Mr. Spencer Wells has recently performed amputation at the hip-joint for malignant growth of the thigh. The patient, a young married woman, recovered from the operation.

*Femoral Aneurism controlled after seven hours digital compression.*—M. Chassaignac communicated to the Surgical Society of Paris the case of a patient affected with femoral aneurism, just above the ring of the adductor magnus. Digital compression was begun at noon, and the tumor was firm and still at seven in the evening, the collateral circulation having taken twelve hours to become established. The compression was exerted on two spots alternately, viz: midway between ilium and pubis, and the muscular masses a little below, the fingers being assisted by a little bag filled with lead, which latter facilitated the manipulations. As the pain was very great in the ilio-pubic, the alternation allowed of continuous compression. The collateral circulation is now well developed, and all the arteries are felt, both in the leg and foot. The compression was carried on for twenty-four hours more, after the condensation of the tumor, in order to prevent a return of the pulsation and insure the permanency of the cure. M. Chassaignac stated that the results of the case had been witnessed by Messrs. Curling, Syme, Taylor and Hodgkin.

*Induction of Premature Labor.*—Henry James, Esq., F. R. C. S., Surgeon and Accoucheur of London and Lying-in Hospital, and Fellow of the Obstetrical Society, has presented seven interesting cases in which he has easily succeeded in inducing premature labor by a very simple process. The patient is placed in the usual obstetric position; fore-finger of the left hand is then passed up to the os uteri, and as soon as possible between its lips, into the uterus; the neck is to be now slightly pulled down and the finger, still within, is to be passed round it as far as possible. Success does not usually attend the first effort; upon repeating this process, however, some two or three times, considerable care being taken not to rupture the membranes, the pains of labor soon come on. The kolpeurynter or elastic bottle, with tube attached, is then passed in; the kolpeurynter is then slowly filled with warm or cold water, producing slow and gradual extension of the os uteri. Labor soon comes on and the delivery is easily effected.

*Legal Evidence of Life in an Infant.*—A case of great interest in medical jurisprudence (case of Brock *versus* Kellock) has recently been decided by the Vice-Chancellor, Sir J. Stuart. The point at issue was to determine the legal evidence of life in an infant.—Dr. Robert Lee and Dr. F. H. Ramsbothem contended that the proof of respiration having been performed was necessary to establish the fact of extra-uterine life. Dr. Tyler Smith, Dr. Freeman and Dr. Alfred Taylor deposed that the continuance of the heart's action after the severing of the umbilical cord must be accepted as proof of independent life. The vice-chancellor, in his decision, confirmed Dr. Tyler Smith's view of the case, and expressed his surprise that a man in Dr. Lee's position should have made such an affidavit. There was a large pecuniary amount involved in the decision. The case is of great importance, as it will serve to establish the law, which has been much unsettled upon the point at issue.

*The Deposit of Tophus in Gout.*—The gouty diathesis is the only one in which the secretion of the tophus is met with. This deposit is composed, in part, of urate of lime; in part, of phosphate of lime. Itsoccurrence has long been known; and in the great "Dictionary of Medicine" may be found the history of a gouty subject, who produced such a quantity of chalk-stone during his lifetime as to supply sufficient material for his own tombstone after death.

*Cure of Gonorrhœa by blisters.*—H. Chalmers Miles has presented a very interesting paper on this subject. By absolute rest, low diet, the administration of mild purgatives and a blister applied either to the corpus spongiosum or to the inside of the thighs, he succeeds in uniformly arresting this disease, and restoring his patient to health in three or four days.

*Radical Cure of Inguinal Hernia.*—Mr. Kingdon, Surgeon to the City of London Truss Society, testifies that the majority of operations for this purpose are ineffectual, inasmuch as the patient is not benefitted for more than a few months. He furnishes seven cases in which Wutzer's ——— operation (the favorite operation with surgeons) had practically failed. The patients had been operated upon by some of the best surgeons in the City of London. There can be only one opinion respecting operative surgery. Its only claim is based upon the permanent benefit which it can effect; and if experience proves that any one operation fails, even at a remote period, in its object, it is the duty of those acquainted with the failure to make the facts public, in order that a proper value may be attached to the proceeding.

*The Advantages of Correct Administration in the Medical Department of an Army.*—After the re-organization of the Medical Department in the British army, in which the establishment of adequate rank and the creation of a thorough system of inspection were the chief features, the reformation produced was as surprising to all concerned as beneficial in its results. Lord Herbert, on moving the thanks of Parliament to the army, for their conduct in China, was able to announce to a cheering House, what no previous War Minister ever had it in his power to say, "that the troops during the whole of that arduous campaign had enjoyed as good a state of health as if they had been at home."

*Pus Cells in the Atmosphere.*—The following discovery has recently been made. In the Orphan Asylum, near Prague, an epidemic of purulent opthalmia lately broke out, and ninety-two children out of two hundred were attacked. Great care was taken to avoid the contact of the matter, but the medical attendants and nurses, nevertheless, took the disease. M. Eiselt thereupon proceeded to examine the air with Pouchet's aëroscope, improved by Purkynje, and in the atmosphere of a ward where lay a great many of the children, a large number of pus cells were found. In fact, the cells were noticed upon the instrument, as soon as the air was made to pass through the apparatus.

*Duration of Life on the Mediterranean Coast.*—It will be observed that, contrary to what is usually supposed, the average duration of life on the Mediteranean coast is far below the standard existing elsewhere. The average duration of life is twenty-nine years at Pisa and Florence, and twenty-eight only at Rome and Naples; whilst at Paris it is thirty-nine, and in London forty-four. For corroborative evidence refer to Dr. Carriere's highly-esteemed work on "*Le Climat de l'Italie.*"

*Mortality in Variola and Varioloid.*—It is gratifying to state that the mortality in these two diseases is not greater in the military hospitals of the Confederacy than in the chief small-pox and vaccination hospitals of England.

In the English hospitals, mortality in variola is 34.78; in Confederate hospitals, mortality in this disease is 40.58. In varioloid, in the English hospitals, the mortality is 9.46; in the Confederate hospitals, the mortality is 3.26. When the many disadvantages attending the construction and establishment of the Confederate small-pox hospitals are considered, this fact is as remarkable as it is gratifying.

*Bloody Sweat.*—In St. Mary's Hospital, London, a well-marked case of this kind has occurred, satisfactorily authenticating the agonies of Gethsemane. It is proper to state that this was a case of vicarious menstruation, occurring in young woman of twenty-seven years of age.

*Pulsation of the Heart after Cessation of Respiration.*—An instance has recently occurred in England where the action of the heart continued fully twenty minutes after all respiration has ceased.

*Special Hospitals.*—These hospitals have been condemned. The report of the Queen's Hospital, Burmingham, endorses the statement, that much detriment to the public and the medical profession arises from the practice of opening small institutions under the name of hospitals for particular forms of disease, in the treatment of which no other management, appliance or attention is required than is to be found in existing general hospitals. It should not be forgotten as a part of the duty of all who protested against the development of special hospitals, that the most efficient arrangements are required for making the treatment of various diseases as complete as possible in General hospitals.

*An unusual Operation.*—M. Kœberte, of Strasburg, has lately performed a rather extraordinary surgical operation. He was engaged in removing, *through* the parietes of the abdomen, a large fibrous tumor of the uterus, when he determined, on perceiving the morbid state of the womb and one of the ovaries, to remove both the latter and the uterus, leaving merely the vaginal portion of the cervix uteri. The patient, five weeks after the adoption of these unusual and severe surgical measures, had experienced neither hemorrhage nor untoward symptoms. She is now (says the *Gazette Médicale de Strasbourg* of the time) quite convalescent.

*The Cause of Diabetes.*—In a case of diabetes, in the service of M. Trousseau, at the Hotel Dieu, he endorses M. Bernard's deductions in regard to the cerebral origin of this disease. It will be remembered that, by pricking certain spots in the walls and floor of the fourth ventricle, M. Bernard succeeded in producing diabetes in animals. In the case of M. Trousseau's patient, the grey, nervous substance, in the neighborhood of the fourth ventricle, was found in a state of fatty degeneration. A similar condition was ascertained to exist in another patient who recently died from diabetes, the details of whose autopsy were presented to the Biological Society by M. Luis.

*Testicle retained in the Inguinal Canal.*—At a meeting of the Pathological Society of London, Mr. Curling showed a specimen of an undescended testicle; in this no spermatozoa were found. The structure was natural, but neither in the testicle nor the vas deferens, nor in the vesicula-seminalis, were there any spermatozoa. Debate elicited the interesting fact, that this is the usual condition in cases of undescended testicle. Mr. Curling also showed the testicles of an idiot; the structure of the testicle was undeveloped. He demonstrated that this condition frequently co-existed with undeveloped brain.

*New Treatment for Pneumonia.*—Dr. Strohl, of the Strasburg School of Medicine, has brought to the attention of the profession the happy effects of the acetate of lead, as a substitute for antimony, in the treatment of this disease. He gives large doses, varying from five to ten grains; the pulse is reduced from ten to fifteen beats per minute, in a very short space of time. No evil effects have resulted from this practice.

*Resection of the Knee-joint in Paris.*—M. Maisonneuve has lately reported a case of this kind, and strongly recommends the operation. He states that he attributes his success to having dressed the wound with spirits of wine.

Mr. Fergusson has lately performed the same operation on a child six years old, in which the recovery was perfect. After healing, the shortening of the limb amounted to no more than an inch.

*An Anomalous Case of Vaccinia.*—Dr. S. Lawrence Gill gives a description, with a plate, of a case of vaccinia in which, instead of one, thirty-two pustules (not vesicles) were formed. The patient was vaccinated in the ordinary manner; the virus being inserted at three points of a triangle; in the centre of each pustule there was a well-marked depression.

*Reward for Gallantry on the Field.*—"Her Majesty has been graciously pleased to confer the decoration of the Victoria Cross, upon Surgeon Herbert Taylor Reade, for conspicuous gallantry during the siege of Delhi. The wounded were in very great danger, when Surgeon Reade drew his sword and calling upon the few soldiers who were near htm, succeeded in driving the enemy from their position."

*Strangulated-oblique Inguinal Hernia, treated by inverting the patient.*—In a well-marked case of this kind, where all methods of relief had been uselessly tried, the difficulty was immediately overcome by inverting the patient. The merit of re-introducing a method of relief which had become obsolete and forgotten, is due to Mr. Jessop, Cheltenham, England.

*The Cisticercus Cellulosus transformed within the Organs of a Man into Tœnia Solium.*—Messrs. Küchenmeister, Leuckart and Humbert have, at last succeeded in proving that the cysticercus cellulosus, when swallowed or received into the system by injection, is transformed into the tœnia solium.

*Chloroform in Puerperal Convulsions.*—Several interesting cases of immediate relief of puerperal convulsions by the administration of chloroform have been reported. The ill effects usually anticipated, were not observed.

*The Impropriety of administering Morphia in Diseases of the Kidneys.*—This fact has been fully established by post-mortem examinations recently made.

*Amputations on the Battle-field.*—By an order of the Surgeon-General of the United States Army, thigh amputations on the battlefield are positively forbidden.

*Ague in an Infant.*—Attack first occurred when the child was ten days old; paroxysms well marked; stages well defined; type, Quotidian; mother had suffered fyom erratic ague for two years.

